# Innovative diagnostic strategies for equine habronemiasis: exploring molecular identification, gene expression, and oxidative stress markers

**DOI:** 10.1186/s13071-025-06970-1

**Published:** 2025-08-02

**Authors:** Mai A. Salem, Sohila M. El-Gameel, Mohamed S. Kamel, Eslam M. Elsamman, Reem M. Ramadan

**Affiliations:** 1 Department of Parasitology, Faculty of Veterinary Medicine, Cairo University, Giza, 12211 Egypt; 2 Department of Medicine and Infectious Diseases, Faculty of Veterinary Medicine, Cairo University, Giza, 12211 Egypt; 3 Faculty of Veterinary Medicine, Cairo University (Equine Veterinarian), Giza, 12211 Egypt

**Keywords:** *Habronema*, PCR, COXI, Oxidative stress, Cytokines, MDA, *IFN-γ*, *TNF-α*

## Abstract

**Background:**

Equine habronemiasis, caused by *Habronema* (*H.*) *muscae*, *H. microstoma*, and *Draschia megastoma*, is a parasitic disease that presents in both gastric and cutaneous forms. Conventional diagnostic methods often lack sensitivity due to intermittent egg shedding and nonspecific clinical signs. This study aimed to enhance diagnostic accuracy by integrating molecular identification, oxidative stress profiling, and cytokine gene expression analysis.

**Methods:**

A total of 100 horses from a private farm in Giza, Egypt, were clinically examined for signs of habronemiasis. Fecal and skin samples were examined using parasitological techniques alongside polymerase chain reaction (PCR) targeting the mitochondrial cytochrome c oxidase subunit I (COXI) gene. The serum levels of oxidative stress biomarkers, including malondialdehyde (MDA), superoxide dismutase (SOD), glutathione (GSH), and total antioxidant capacity (TAC), were quantified. Additionally, the expression of cytokines (interferon-gamma (*IFN-γ*), tumor necrosis factor-alpha (*TNF-α*), interleukin 1 beta (*IL-1β*) and interleukin-6 (*IL-6*))was assessed via real-time PCR.

**Results:**

*Habronema* spp. eggs were detected in 62% of fecal samples, with molecular analysis confirming *H. muscae* as the predominant species. Infected horses exhibited significantly elevated oxidative stress markers compared with those in healthy controls. Cytokine gene expression analysis demonstrated a marked upregulation of proinflammatory markers, indicating a Th1-dominated immune response.

**Conclusions:**

This study underscores the value of molecular diagnostics combined with immunological profiling for the detection and characterization of equine habronemiasis. The integration of oxidative stress and cytokine biomarkers provides important insights into host–pathogen interactions and may contribute to the development of improved diagnostic and therapeutic strategies.

**Graphical Abstract:**

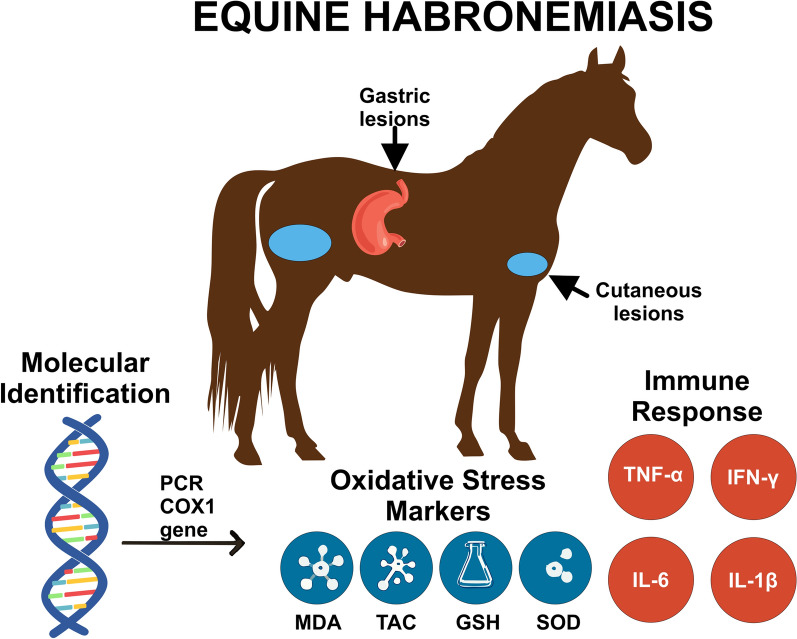

**Supplementary Information:**

The online version contains supplementary material available at 10.1186/s13071-025-06970-1.

## Background

Habronemiasis is a parasitic disease in equines caused by nematodes of the genus *Habronema*, specifically *Habronema* (*H.*) *muscae*, *Habronema microstoma*, and *Draschia megastoma*. These parasites possess a complex life cycle that involves muscid flies, particularly *Musca domestica* and *Stomoxys calcitrans*, as intermediate hosts [1]. Infected adult worms typically reside in the equine stomach, resulting in gastric habronemiasis, while their larvae can aberrantly migrate to the cutaneous tissues and cause cutaneous habronemiasis [2].

Gastric habronemiasis typically manifests as catarrhal gastritis, presenting with symptoms such as diarrhea, progressive weight loss, and gastric ulcers [3]. However, traditional diagnostic methods, such as fecal examinations, often lack sensitivity because of the intermittent shedding of eggs and the morphological similarity of *Habronema* eggs to those of other nematodes [2, 4]. To overcome these limitations, advancements in molecular diagnostics have played a significant role. In particular, semi-nested polymerase chain reaction (PCR) assays targeting ribosomal DNA (rDNA) markers or mitochondrial genes have greatly improved the detection of *H. microstoma* and *H. muscae* DNA in equine feces. These methods offer enhanced sensitivity, enabling reliable identification even in subclinical infections or cases with low parasite burdens [5, 6].

Cutaneous habronemiasis, also known as summer sores, develops when infective larvae are deposited onto moist areas or wounds, resulting in granulomatous skin lesions characterized by ulceration and pruritus [7]. These lesions often prove refractory to standard treatments, making an accurate diagnosis essential [8]. In addition, recent studies have highlighted the involvement of inflammatory and oxidative stress markers in the pathogenesis of cutaneous habronemiasis. Notably, the levels of oxidative stress markers, such as malondialdehyde (MDA), are significantly elevated, indicating oxidative damage. In addition, increased levels of proinflammatory cytokines, such as interleukin 6 (*IL-6*) and tumor necrosis factor alpha (*TNF-α*), have been observed in affected horses [9].

Parasitic infections cause varying degrees of stress in the host, including oxidative stress, as the immune system produces reactive oxygen species (ROS) to combat the invasive pathogens. However, ROS cannot distinguish between infectious agents and host cells, resulting in potential collateral damage [10]. Oxidative stress occurs when the production of ROS exceeds the host antioxidant defenses [11]. This overproduction can lead to oxidative changes in essential cellular components, such as DNA, proteins, and lipids. For example, ROS-induced lipid peroxidation damages cell membranes, causing a loss of selective permeability and increased cellular injury. Similarly, oxidative DNA damage may result in mutations, replication errors, genomic instability, and cell death, whereas oxidative damage to proteins can impair critical metabolic activities [12].

The cell-mediated immune response is crucial for defending the host against parasitic infections by limiting pathogen multiplication, although it may also contribute to tissue pathology. T helper 1 (Th1) cytokines, particularly *IFN-γ*, play a central role during the early acute stages of parasitic disease [13]. *IFN-γ* is essential for inducing host immunity by stimulating monocytes to enhance microbicidal activity and regulating immunoglobulin (Ig)G2 production from bovine B cells. Along with* IFN-γ, TNF-α* serves as another key proinflammatory cytokine that mediates resistance against intracellular parasites by enhancing phagocytic activity and supporting granuloma formation. Additionally, *TNF-α* acts synergistically with *IFN-γ* to inhibit parasite replication and promote parasite clearance. However, excessive or prolonged *TNF-α* production can contribute to immunopathology and tissue damage [14]. This Th1-dominated response is part of a broader immunological shift characterized by an antagonistic balance with Th2 cytokines. The primary immune mechanism against many parasitic infections remains cell-mediated, as mononuclear cells such as lymphocytes and macrophages release cytokines including *IFN-γ, TNF-α*, and various interleukins (*ILs*) to suppress parasite growth and dissemination. Despite the critical role of these inflammatory mediators in shaping disease outcomes, few studies have specifically evaluated their role in equine habronemiasis [15]. Therefore, elucidating the immune components involved in host–parasite interactions is essential for broadening our understanding of the molecular mechanisms at the host–pathogen interface. This knowledge is vital for identifying new targets that could aid in developing preventive strategies to block pathogen transmission.

This study aimed to comprehensively investigate *Habronema* infections in horses by integrating molecular, immunological, and biochemical diagnostic approaches. Specifically, this study sought to determine the prevalence of *Habronema* species and characterize them using molecular identification techniques. Phylogenetic analysis based on mitochondrial cytochrome c oxidase subunit 1 gene sequencing was performed to confirm species identity and to explore genetic variation. In addition to molecular diagnostics, this study analyzed the gene expression of immune signaling pathway components involved in *Habronema* infection in horses. Furthermore, oxidative stress and immunological markers were assessed to evaluate their significance in infected equines. Collectively, this integrated diagnostic strategy was designed to enhance our current understanding of equine habronemiasis.

## Methods

### Study area, animal selection and management

This study was conducted from April to October 2024 at a private equine farm located in Nazlet El-Saman, Giza, Egypt (latitude: 30.013056, longitude: 31.208853). The farm specializes in breeding and managing light-riding Arabian horses. A total of 100 horses of different ages and sexes were randomly selected and examined for *Habronema* spp. using clinical and parasitological examinations. The horses had a mean age of 6.2 ± 2.8 years and an average body weight of 420 ± 35 kg. The animals were housed under uniform management practices throughout the study period. All horses received balanced rations consisting of hay (hay cubes or hay-based pellets) at 1% of body weight and concentrates (barley and corn grains) at 1.5–2% of body weight, with additional feed items such as carrots, vegetable clover, dried whey, and linseed provided as supplements to the diet. Commercial products included Tribute^®^ High Fat Pelleted Feed (13% fat, 20% fiber, and 15% starch/sugar) and Biotinvet 10,000 (EQUIPLANET, Italy), a dietary mineral supplement. Clean drinking water was provided ad libitum. Routine vaccinations for foals were administered at 3 months of age against equine influenza and equine herpesvirus. Veterinary care on the farm was sought only when health issues were observed by the owner, and previous records indicated a seasonal increase in *Musca* spp. associated conditions during the warmer months.

### Parasitological sampling and examination

The farm remained under continuous parasitological monitoring for six successive months, during which fecal and blood samples were collected monthly from each selected horse. Fecal samples were obtained rectally using sterile gloves and examined within hours of collection. Standard parasitological techniques, including flotation and sedimentation, were used to identify *Habronema* spp. eggs [7]. In horses exhibiting cutaneous lesions, visual inspection and superficial skin scrapings were performed to confirm cutaneous habronemiasis [9]. Blood samples were collected via jugular venipuncture using sterile vacutainer tubes. All collected samples were immediately transported under controlled conditions to the Parasitology Laboratory at the Faculty of Veterinary Medicine, Cairo University, for further processing and analysis. Eggs were identified according to the methods described by Devi et al. [7].

### Molecular identification

Molecular analysis was performed to detect and characterize *Habronema* spp. from both gastric and cutaneous cases in horses. Fecal samples were collected rectally using sterile gloves and immediately preserved in 70% ethanol to stabilize the DNA. Microscopic screening, including flotation and sedimentation techniques, was performed to identify the presence of *Habronema* eggs. DNA was extracted from positive fecal samples using a commercial fecal DNA extraction kit, ensuring suitability for downstream PCR analysis. In cases suspected of cutaneous habronemiasis, clinical samples were obtained from ulcerative skin lesions by taking sterile punch biopsies from the lesion margins after local anesthesia, along with deep skin scrapings and exudate swabs from the lesions. All cutaneous materials were placed in sterile tubes containing 70% ethanol and transported on ice to the laboratory for further molecular processing. Genomic DNA was then extracted according to the manufacturer’s tissue protocol using the QIAamp DNA Mini Kit (Qiagen, Germany). The purity and concentration of the extracted DNA were measured using a Nanodrop2000 spectrophotometer (NP80, Nanophotometer, Implen, Germany). PCR amplification targeted the COXI gene (689 base pairs) using specific degenerate primer pairs (forward (F): 5′-TGATTGGTGGTTTTGGTAA-3′ and reverse (R): 5′-ATAAGTACGAGTATCAATATC-3′) developed by Iorio et al. [16]. PCR reactions were set up in 25-μl volumes with Emerald Amp Max PCR Master Mix (Takara, Japan), and thermal cycling conditions were slightly modified from Ramadan et al. [17, 18]: initial denaturation at 94 °C for 7 min, followed by 40 cycles at 94 °C for 1 min, 50 °C for 1 min, and 72 °C for 1 min, with a final extension at 72 °C for 10 min. Amplicons were purified using a QIAquick PCR purification kit (Qiagen, USA). The COXI PCR products were sent to Macrogen Inc. (Seoul, South Korea) for bidirectional sequencing using the same primer pairs; sequencing was performed with the Big Dye terminator cycle sequencing kit and analyzed on a 3730XL sequencer (Applied Biosystems™, USA). The raw sequence data were edited and assembled using the BioEdit program [19] and then submitted to GenBank for accession number assignment. The assembled sequences were compared with existing entries using the National Center for Biotechnology Information Basic Local Alignment Search Tool (NCBI BLAST) [20]. The sequences of the mitochondrial COXI gene from *H. muscae* and related nematode species were retrieved from NCBI GenBank, and multiple sequence alignment was performed with MAFFT version 7. Phylogenetic trees were constructed using MEGA X software via the maximum likelihood (ML) method with bootstrap analysis of 1,000 replicates to estimate evolutionary distances; branch lengths reflect the number of substitutions per site. The final phylogenetic analysis included sequences from *H. muscae*, *Parabronema skrjabini*, *Wuchereria bancrofti*, *Setaria tundra*, *Dirofilaria* spp., and *H. majus*.

### Measurement of oxidative stress markers

The serum samples were evaluated for several indicators of oxidative stress, including superoxide dismutase (SOD), malondialdehyde (MDA), glutathione (GSH), and total antioxidant capacity (TAC). These parameters were measured in both positive and negative sera of the examined horses using specialized kits, as described by Attia et al. [21] and Salem et al. [22].

### Cytokine expression quantification

Total RNA was extracted from the buffy coats of all infected horses using 8.5 µl of sterile TRIzol Reagent (Invitrogen Life Technologies, Carlsbad, CA, USA) and 10 pmol of Metabion, International AG, following the manufacturer’s recommendations. Additionally, the total RNA was extracted from the buffy coats of five clinically healthy horses designated as negative controls. These horses were rigorously confirmed to be free of bacterial, viral, parasitic, and fungal infections through comprehensive clinical and parasitological evaluations and molecular diagnostics, including PCR assays, to exclude subclinical infections. All negative control animals were maintained on the same farm to ensure comparable environmental exposure and management conditions, thereby minimizing the potential confounding factors. In addition, the positive controls included samples obtained from horses with confirmed *Habronema* infection. Infection was confirmed through clinical evaluation, parasitological examination, and PCR amplification of the COXI gene. These positive control samples consistently exhibited upregulation of proinflammatory cytokine genes, including *TNF-α, IFN-γ, IL-1β*, and *IL-6*.

A total of 2 µl of template RNA was used for downstream applications. To assess RNA quantity and integrity, an aliquot of total RNA diluted in RNase-free water was set aside, while the remaining sample was stored at −80 °C until gene expression analysis. The RNA concentration and purity were determined using a Nano-Drop ND-1000 Spectrophotometer (Nano-Drop Technologies Inc., Delaware, USA) as described by Salem et al. [23].

Relative quantitative reverse transcription polymerase chain reaction (qRT-PCR) was performed to measure the expression of cytokine genes, including *TNF-α, IFN-γ, IL-1β*, and *IL-6*. Primers specific to equine sequences were designed on the basis of GenBank entries (Table 1). The glyceraldehyde-3-phosphate dehydrogenase (*GAPDH*) was used as an internal reference for normalization. Relative gene expression was calculated using the comparative Ct method (2^−ΔΔCt^), as described by Livak and Schmittgen [24]. Noninfected horses served as negative controls to establish the baseline expression levels of each gene.

**Table 1:** Oligonucleotide primers for qRT-PCR

Genes	Primers sequences	References
*IFN-γ*	F-TCTTTAACAGCAGCACCAGAA R-GCGCTGGACCTTCAGATCAT	[17]
*IL-1β*	F- AAAACAGTGAGGGAGAAATT R- AGAAACTTCTTCTTGGGTAG	[27]
*IL-6*	F-GGATGCTTCCAATCTGGGTTCAAT R-TCCGAAAGACCAGTGGTGATTTT	[28]
*TNF-α*	F-ATGTTTCAGTCACATTTCAG R-CCTACCGGTTCCCATCTCAA	[27]
*GADPH*	F-CCTGGAGAAACCTGCCAAG R-GCCAAATTCATTGTCGTACC	[17]

F, forward primer; R, reverse primer

 Reverse transcription polymerase chain reaction (RT-PCR) assays were performed using the Cepheid SmartCycler^®^ II system (Sunnyvale, CA, USA). Each reaction had a final volume of 25 µl, containing 0.5 µl of each primer (10 pmol), 12.5 µl of SYBR Green PCR master mix (Applied Biosystems, USA), 1 µl complementary DNA (cDNA) (400 ng), and 10.5 µl RNase-free water [25]. The amplification protocol consisted of an initial incubation at 95 °C for 5 min, followed by 40 cycles of denaturation at 95 °C for 30 s, annealing at 60 °C for 30 s, and extension at 60 °C for 30 s for *IFN-γ, IL-6, IL-1β*, and *TNF-α* [26].

### Statistical analysis

Oxidative stress markers and gene expression data were analyzed using SPSS software (version 27, SPSS Inc., Chicago, IL, USA) [29]. The Shapiro–Wilk test was used to evaluate the normality of the datasets, with *P*-values greater than 0.05 indicating a normal distribution. Group comparisons were performed using one-way analysis of variance (ANOVA). When ANOVA revealed significant differences, Tukey’s post hoc test was conducted to determine which groups differed. Statistical significance was set at *P* < 0.05 [30]. The results are shown as mean ± standard deviation (SD). The levels of significance are as follows: *P* < 0.05 (*), *P* < 0.01 (**), *P* < 0.001 (***), and *P* < 0.0001 (****).

## Results

### Clinical signs of inspected equines

Out of the 100 horses examined, a variety of clinical signs of habronemiasis were observed; 13 horses (13%) exhibited both cutaneous and gastric forms of infection. Cutaneous lesions are characterized by skin papules, excessive granulation tissue (proud flesh), pruritus, and nonhealing wounds, often containing small yellow calcified granules. These lesions are typically solitary and most commonly appear on the face (below the eye), neck, ventral abdomen, and limbs. The clinical symptoms associated with cutaneous habronemiasis included ulcerations, exudation, intermittent bleeding, swelling, itching, and occasional secondary bacterial infections (Supplementary Material Fig. S1). By contrast, 49 horses (49%) presented with clinical signs consistent with gastric habronemiasis only, such as intermittent colic, poor body condition, inappetence, dull hair coat, and weight loss. The remaining 38 horses (38%) were negative for both cutaneous and habronemiasis.

### Microscopic examination of fecal samples

A total of 62 samples (62%) tested positive for *Habronema* spp. eggs. The observed eggs were elongated, thinly shelled, and embryonated, displaying morphological characteristics consistent with those of *Habronema* species. These parasitological findings were closely correlated with clinical observations, particularly in horses presenting with signs of gastric discomfort.

### Molecular detection of *Habronema* species involved in cutaneous and gastric habronemosis

**Fig. 1:**
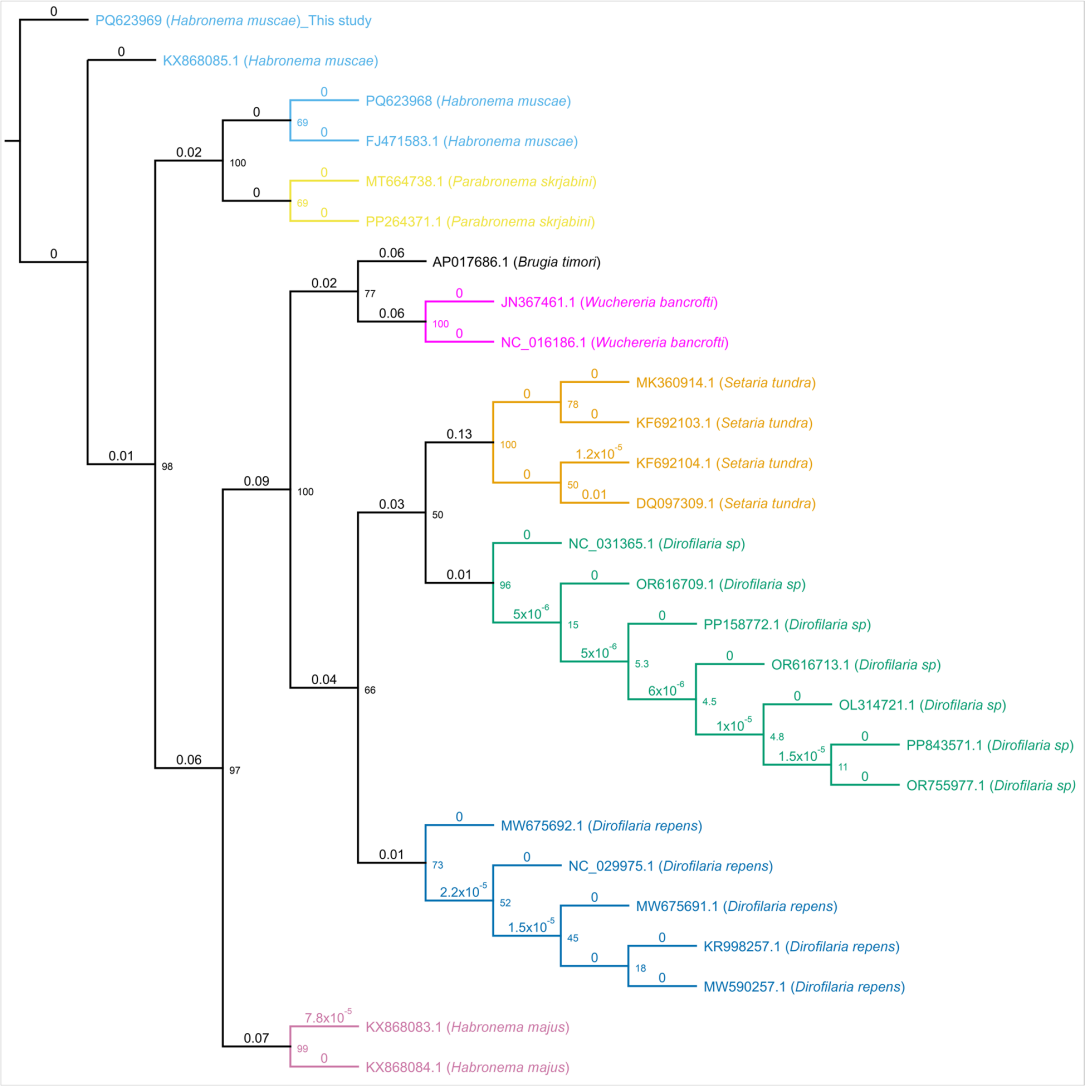
Maximum likelihood phylogenetic tree based on partial COXI gene sequences illustrating the relationships among *H. muscae* isolates obtained in this study and related nematode species. Bootstrap values (1,000 replicates) are shown at nodes. Branch lengths are proportional to the number of nucleotide substitutions per site. GenBank accession numbers are indicated

The ML phylogenetic tree revealed that the sequences obtained in this study clustered tightly with the reference sequences of *H. muscae* (GenBank accession numbers KX868085.1, FJ471583.1, and PQ623968), confirming their species identity with high bootstrap support (Fig. 1). This cluster was distinct and well-separated from closely related nematode taxa, including *Parabronema skrjabini*, which formed a sister clade. The tree also showed clear separation of other filarial nematodes, such as *Wuchereria bancrofti*, *Setaria tundra*, and various *Dirofilaria* species, each forming monophyletic clades with strong bootstrap values.

**Fig. 2:**
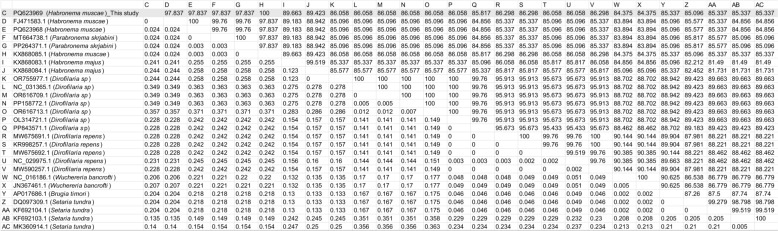
Pairwise sequence similarity matrix showing percentage nucleotide identity among partial COXI sequences of *H. muscae*, related nematodes, and outgroup species. The matrix highlights high within-species similarity among *H. muscae* sequences (> 97%) and clear genetic divergence from other genera such as *Parabronema*, *Wuchereria*, and *Dirofilaria*. GenBank accession numbers are listed for each sequence

Pairwise sequence similarity analysis (Fig. 2) demonstrated high nucleotide identity (> 97%) within the *H. muscae* group, whereas interspecific similarity with related taxa such as *Parabronema skrjabini* and *H. majus* was lower, ranging between 83% and 85%. These results confirm the genetic distinctness of the *H. muscae* isolates analyzed in this study compared with those of other nematode species.

### Oxidative stress of *H. muscae* in infected horses

**Fig. 3:**
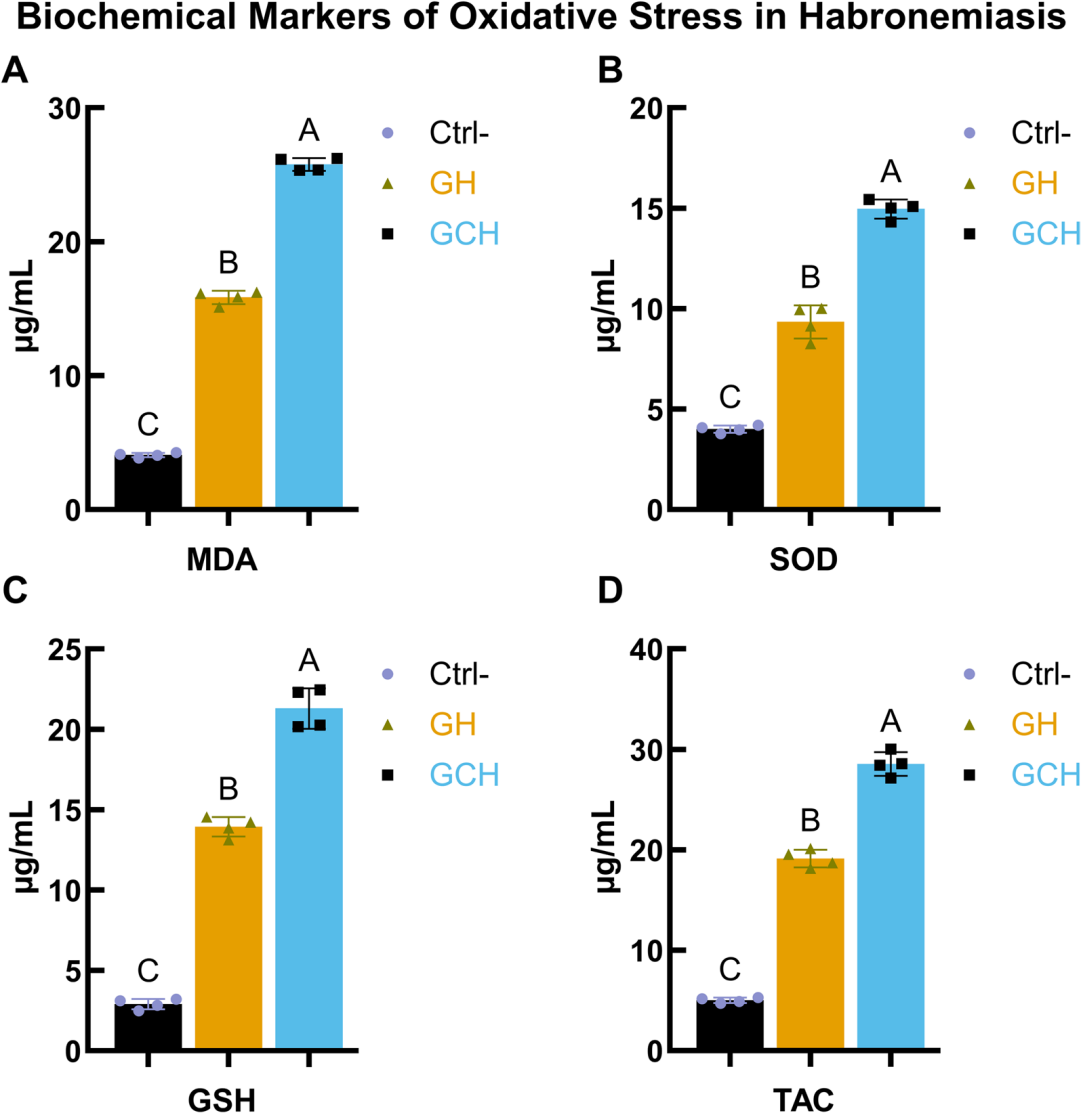
Biochemical markers of oxidative stress in habronemiasis: levels of MDA, SOD, GSH, and TAC. Biochemical markers of oxidative stress **A** malondialdehyde (MDA), **B** superoxide dismutase (SOD), **C** glutathione (GSH), and **D** total antioxidant capacity (TAC) measured in control (Ctrl-), gastric habronemiasis (GH), and cutaneous gastric habronemiasis (GCH) groups. The values are expressed as mean ± SD. Different letters (A, B, C) indicate significant differences between groups (*P* < 0.05)

Compared with those in healthy controls, oxidative stress markers were significantly altered in animals affected by habronemiasis. Malondialdehyde (MDA), an indicator of lipid peroxidation, was elevated in both the gastric habronemiasis (GH) and cutaneous gastric habronemiasis (GCH) groups, with the highest levels observed in the GCH group (Fig. 3A). SOD activity increased significantly in the GH group and was further increased in the GCH group compared with the control group (Fig. 3B). Similarly, the glutathione (GSH) concentration was significantly greater in the GH and GCH groups than in the control group, with the greatest increase detected in the GCH group (Fig. 3C). TAC also followed this trend, showing a significant increase in both infected groups compared with the control groups (Fig. 3D). These findings indicate that habronemiasis induces oxidative stress, accompanied by a compensatory increase in antioxidant defenses, which is more pronounced in GCH.

### Immune response of *H. muscae* on infected horses

**Fig. 4:**
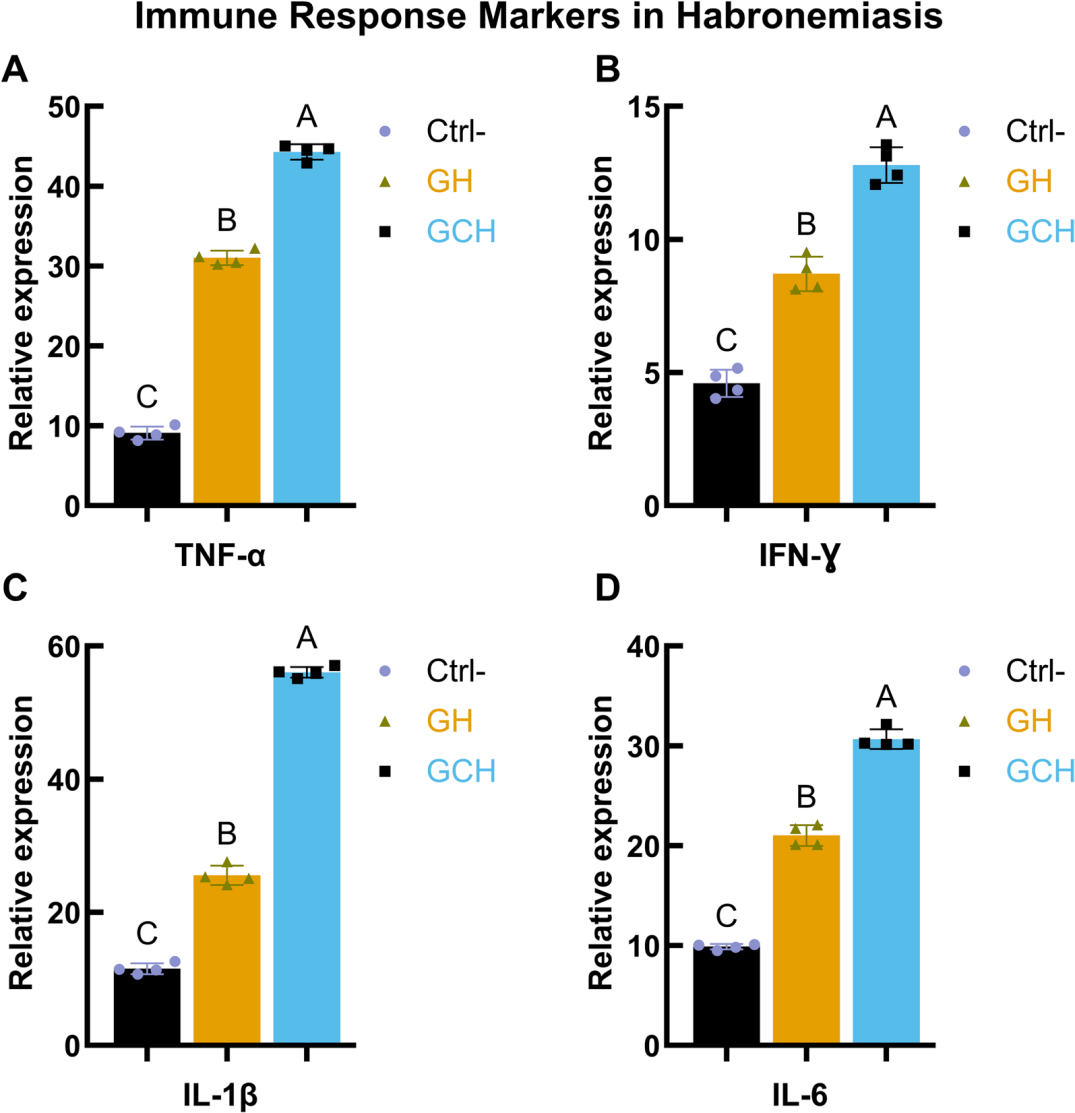
Immune response markers in habronemiasis: levels of *TNF-α, IFN-γ, IL-1β*, and *IL-6*. Concentrations of **A** tumor necrosis factor alpha (*TNF-α*), **B** interferon gamma (*IFN-γ*), **C** interleukin 1 beta (*IL-1β*), and **D** interleukin 6 (*IL-6*) in control (Ctrl-), gastric habronemiasis (GH), and cutaneous gastric habronemiasis (GCH) groups. Immune marker levels were measured using qPCR, with gene expression normalized to *GAPDH* and calculated by the 2^−ΔΔCt^ method. The data are presented as mean ± SD for each group. Different letters (A, B, C) indicate statistically significant differences between groups (*P* < 0.05)

Compared with those in the control animals, immune response markers were upregulated in the animals infected with habronemiasis. Tumor necrosis factor alpha levels were significantly increased in the GH group and were highest in the GCH group (Fig. 4A). Similarly, compared with those in the controls, *IFN-γ* concentrations were significantly greater in the GH group and in the GCH group (Fig. 4B). The proinflammatory cytokine *IL-1β* exhibited a similar pattern, with elevated levels of GH and the greatest increase in GCH (Fig. 4C). *IL-6* levels were also significantly elevated in both infected groups compared with those in the control group (Fig. 4D). These results suggest an enhanced inflammatory immune response associated with habronemiasis, which is more pronounced in the cutaneous form.

## Discussion

Habronemiasis is a common equine parasitic disease caused by *Habronema* spp., primarily *H. muscae*, *H. microstoma*, and *Draschia megastoma*. The disease manifests in both gastric and cutaneous forms. The gastric form involves adult worms inhabiting the stomach, resulting in gastritis and weight loss. By contrast, the cutaneous form, referred to as “summer sores,” arises when larvae are deposited on wounds, leading to ulcerative and granulomatous skin lesions [2, 3]. The transmission of the parasite occurs through muscid flies, namely *Musca domestica* and *Stomoxys calcitrans*, which serve as intermediate hosts during warm seasons [1]. In this context, the present study offers a comprehensive evaluation of equine habronemiasis by integrating clinical, molecular, and immunological approaches, thereby providing novel insights into host–parasite dynamics specific to *H. muscae* infection. Our results revealed a significant prevalence of both cutaneous and gastric habronemiasis in the horses examined, with molecular diagnostics confirming *H. muscae* as the predominant species.

Traditional diagnostic methods based on clinical signs and fecal microscopy remain widely used but are limited by the intermittent shedding of parasite eggs and their morphological similarities with other strongyle-type ova [1, 2], which can hinder accurate diagnosis. By contrast, PCR amplification targeting the mitochondrial cytochrome c oxidase subunit I gene demonstrated high specificity and sensitivity for definitive species identification, consistent with the findings of previous molecular studies [6, 16]. Sequences obtained in this study showed greater than 97% identity with *H. muscae* sequences in GenBank, and phylogenetic analysis grouped these isolates with known *H. muscae* references with strong bootstrap support, suggesting a conserved genetic makeup with limited geographical divergence [6, 31]. The molecular approach distinguishes *H. muscae* from closely related nematodes, such as *H. microstoma* and *Parabronema skrjabini*, which is essential in endemic regions where overlapping infections may occur [2]. Moreover, fly vectors, such as *Musca domestica* and *Stomoxys calcitrans*, play pivotal roles in transmission by depositing larvae into wounds or mucous membranes during warm seasons [1]. This underscores the importance of timely and accurate identification through PCR, which can help break the transmission cycle by enabling informed treatment and implementing preventive measures.

The low intraspecific genetic variability observed among the *H. muscae* isolates in this study suggests a conserved genetic structure within the local parasite population, mirroring findings from equine populations in Italy and China, which indicate the limited geographic divergence of *H. muscae* [6, 31]. However, broader investigations involving a greater number of isolates and geographic regions are necessary to explore potential strain-specific virulence or resistance patterns. Overall, the molecular methods employed in this study provide rapid, sensitive, and species-specific diagnoses that outperform conventional microscopy, particularly for early or subclinical infections. The application of such tools in field settings could greatly enhance epidemiological surveillance and support more effective targeted treatments and control strategies.

The present study revealed significant changes in the blood markers of oxidative stress in horses infected with *Habronema*. These findings align with the host’s efforts to develop intricate defense mechanisms in response to parasitic infection, particularly through the generation of ROS and/or nitrogen species. Notably, SOD produces hydrogen peroxide, which serves as a substrate for MDA. However, excessive hydrogen peroxide can be harmful to cells. Our investigation revealed elevated levels of MDA, SOD, GSH, and TAC activity in infected horses compared with healthy horses, suggesting that oxidative stress results from an imbalance between increased blood markers and the body’s scavenging mechanisms. In response to this imbalance, the host activates various antioxidant defenses; however, if ROS production overwhelms these systems, oxidative stress occurs [11]. Overproduction of ROS can cause oxidative changes in DNA, proteins, and lipids, which are essential cellular components. For instance, ROS-induced lipid peroxidation compromises cell membrane permeability and increases cellular damage, whereas oxidative DNA damage may lead to mutations, replication errors, genomic instability, and cell death. Furthermore, oxidative damage to proteins can result in the loss of important metabolic activity [12]. Antioxidants such as GSH and SOD are vital for mitigating these harmful effects, especially as the rate of parasitemia increases with their presence. Specifically, SOD acts as an efficient catalyst and demonstrates high resistance to oxidative stress [32], highlighting the importance of the interplay between ROS generation and antioxidant defense in the pathophysiological responses of horses infected with *Habronema*.

This is the first study to evaluate the reactions to *Habronema* infection with a specific focus on the gene expression of *IFN-γ, IL-6, IL-1β*, and *TNF-α*. The results showed a significant elevation in the levels of these cytokine parameters in horses infected with *Habronema* compared with noninfected control animals, and this increase was directly correlated with *Habronema* infection. Notably, this study marks the first investigation in Egypt to examine cytokine responses to *Habronema*, providing valuable regional insights. These findings are consistent with those of previous studies by Maia and Campino [33] and Sasindran and Torrelles [34], who reported elevated cytokine mRNA levels in the context of bacterial and parasitic infections. Furthermore, this study demonstrated that *Habronema* infection in horses leads to significantly increased cytokine expression, contributing new evidence regarding host–parasite interactions. Overall, this study offers important new insights into the mechanisms involved in controlling *Habronema* infection and advances our understanding of the immunological responses associated with this parasitic disease [35].

While this study provides valuable insights into the diagnosis and immunological characterization of equine habronemiasis, it is important to recognize certain limitations inherent to this field-based research. Although the single-farm setting may limit broad generalizability, the comprehensive integration of clinical, molecular, biochemical, and immunological data strengthens the reliability of our findings within this context. Focusing on the mitochondrial COXI gene allowed for precise species identification; however, future studies incorporating additional genetic markers could provide a more detailed understanding of parasite diversity. The cross-sectional nature of this study provides an important snapshot of infection dynamics, and longitudinal research could further elucidate the temporal changes in immune responses and disease progression. Despite these constraints, the significant alterations detected in oxidative stress and cytokine profiles highlight key host–pathogen interactions that warrant deeper investigation. Moving forward, expanding molecular surveillance across diverse populations, developing rapid diagnostic tools on the basis of molecular and biomarker signatures, and exploring targeted vector control strategies will be essential for improving equine health management.

## Conclusions

This study developed an integrated diagnostic platform for equine habronemiasis, combining molecular, biochemical, and immunological tools to achieve sensitive and specific detection of *H. muscae* infections. PCR amplification of the mitochondrial COXI gene allowed precise species identification, surpassing the limitations of traditional microscopy. Phylogenetic analysis confirmed the genetic distinctness of the local isolates, with minimal intraspecific variation. Oxidative stress profiling revealed significant increases in MDA, SOD, GSH, and TAC in infected horses, reflecting an active host defense mechanism against parasitic burden. Gene expression analysis further demonstrated marked upregulation of proinflammatory cytokines—*TNF-α, IFN-γ, IL-1β*, and *IL-6*—indicating a Th1-skewed immune response that may contribute to both parasite control and tissue pathology. This comprehensive approach offers several advantages: (i) accurate molecular identification of *H. muscae*, (ii) simultaneous assessment of oxidative and immune biomarkers, (iii) enhanced understanding of host–pathogen interactions, (iv) the potential to improve early diagnosis, (v) support for targeted treatment strategies, and (vi) applicability in endemic regions with limited diagnostic resources. Despite several limitations related to its geographic scope, the platform provides a robust framework that can be expanded through further validation and adaptation. Overall, this integrated diagnostic strategy holds promise for improving the management and control of equine habronemiasis by reducing misdiagnoses and enabling more informed interventions.

## Supplementary Information

Supplementary material 1.

## Data Availability

Data supporting the main conclusions of this study are included in the manuscript
