# Supplementary material for: Innovative diagnostic strategies for equine habronemiasis: exploring molecular identification, gene expression, and oxidative stress markers
Source: Parasit Vectors. 2025 Aug 2;18:325. doi: 10.1186/s13071-025-06970-1 (PMC12318383; doi:10.1186/s13071-025-06970-1)
Supplement: Supplementary file 1 — Supplementary material 1. [file 13071_2025_6970_MOESM1_ESM.docx]

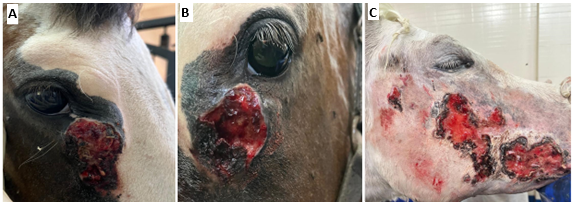


Fig. S1. Horses exhibiting various clinical signs of cutaneous habronemosis: (A-B) prominent *Habronema* lesions on the face, particularly beneath the eye, and (C) a lesion extending along the entire side of the face.
